# Typical ictal pattern on MR perfusion scan for patients on the ictal–interictal continuum

**DOI:** 10.1002/epd2.70078

**Published:** 2025-08-06

**Authors:** Melissa Huynh Mabry, Hrishikesh Dadhich, Zerrin Yetkin, Irfan Sheikh

**Affiliations:** ^1^ Epilepsy Section, Department of Neurology University of Texas Southwestern Medical Center Dallas Texas USA; ^2^ Division of Neuroradiology, Department of Radiology University of Texas Southwestern Medical Center Dallas Texas USA; ^3^ Peter O'Donnell Jr. Brain Institute University of Texas Southwestern Medical Center Dallas Texas USA

A 59‐year‐old man with a history of intraparenchymal hemorrhage presented in status epilepticus with new onset left temporal electrographic seizures. Due to continued seizure activity, he received levetiracetam, lacosamide, clobazam, and a midazolam infusion. Afterwards, the EEG showed continuous 1–2 Hz lateralized periodic discharges (LPDs), maximal at P7/O1, consistent with the ictal‐interictal continuum (IIC) (Figure 1). Brain MRI on admission showed post‐ictal changes in the left temporo‐occipital cortex and left thalamus, and chronic left temporal encephalomalacia. After the patient remained in IIC for 8 days, MR perfusion showed increased relative cerebral blood flow and blood volume in the left temporo‐occipital lobe concerning ongoing ictal activity (Figure 2). Despite treatment, the patient's mental status did not improve and passed away after withdrawal of care. MR perfusion can help determine if an IIC pattern is more ictal than interictal and may alter clinical management.^1^, ^2^, ^3^, ^4^, ^5^

**FIGURE 1:**
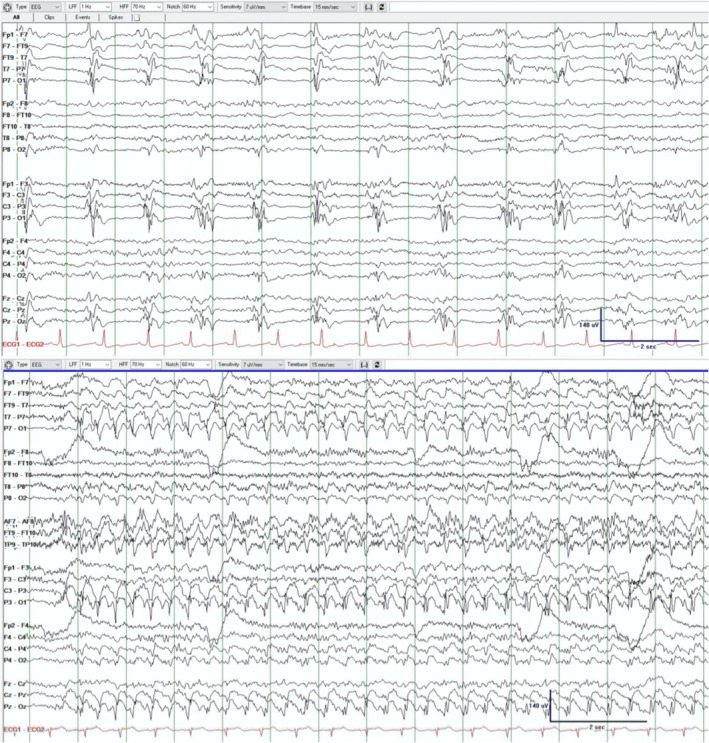
EEG demonstrating left temporo‐occipital LPDs (top panel) and electrographic seizure (bottom panel) thus highlighting the patient's background when in IIC versus when ictal. The electrographic seizure shown was recorded after the MR perfusion scan was performed.

**FIGURE 2:**
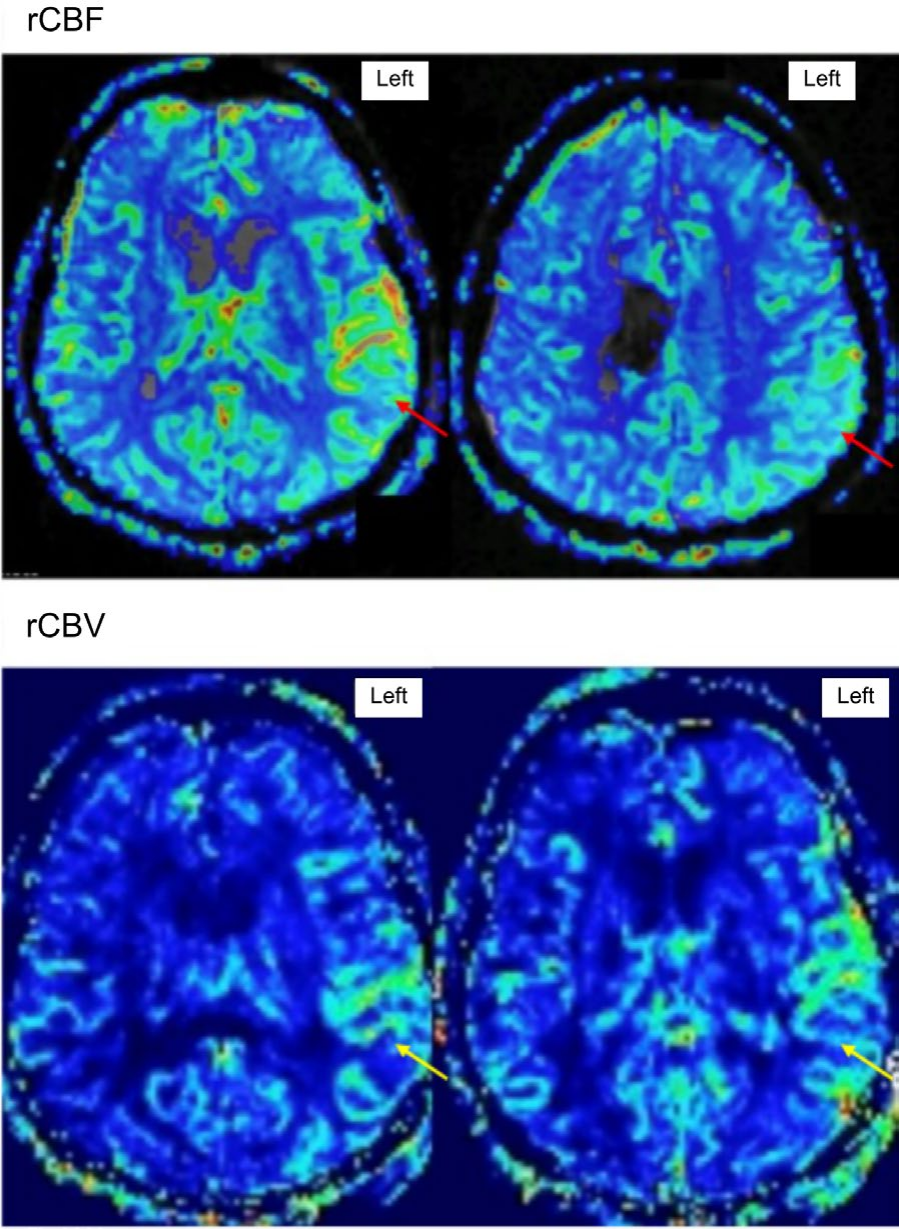
Dynamic susceptibility contrast‐enhanced MR perfusion images demonstrate increased relative cerebral blood flow (rCBF; red arrows) and relative cerebral blood volume (rCBV; yellow arrows) of the left hemisphere. The increased perfusion, particularly in the left temporal lobe, indicated ongoing ictal activity despite the last electrographic seizure occurring 8 days earlier.

## FUNDING INFORMATION

This study was not funded.

## CONFLICT OF INTEREST STATEMENT

Dr. Irfan Sheikh is an associate editor for the Epileptic Disorders Journal and is Assistant Program Leader for the EPD Internship Program. All other listed authors have no conflicts of interest to disclose.

## PATIENT CONSENT

Patient consent was unable to be collected due to the patient being intubated at the time of his admission and subsequently deceased with no family or next of kin available in contact to attain consent. The study was approved by the UTSW IRB as IRB Exempt status STU27031.

**Test yourself**

1. Which of the following electrographic patterns would qualify as part of the ictal‐interictal continuum (IIC)?
  - Lateralized periodic discharges that average 0.5 hz for 10 s
  - Lateralized periodic discharges that average 2.5 hz for 5 s
  - Lateralized periodic discharges that average 2.5 hz for 10 s
  - Lateralized periodic discharges that average 4 hz for 5 s
  - Lateralized periodic discharges that average 4 hz for 10 s
2. Which of the following best describes a typical finding on MR perfusion during the ictal phase of a seizure?
  - Decreased cerebral blood flow and volume in the seizure focus
  - Increased cerebral blood flow and volume in the seizure focus
  - Normal perfusion with no changes in cerebral blood flow or volume
  - Increased cerebral perfusion in the bilateral occipital lobes
  - Global hypoperfusion affecting all brain regions equally
3. Which of the following best describes a typical finding on MR perfusion during the postictal state after a seizure?
  - Hypoperfusion in the region of the seizure focus
  - Symmetric hyperperfusion in both temporal lobes
  - Increased perfusion in the brainstem
  - Hyperperfusion in the contralateral hemisphere
  - Global cerebral hyperperfusion

*Answers may be found in the* *supporting information*

## Supporting information


Data S1.

Data S2.

## Data Availability

Data sharing is not applicable to this article as no new data were created or analyzed in this study.
