# Supplementary material for: Typical ictal pattern on MR perfusion scan for patients on the ictal–interictal continuum
Source: Epileptic Disord. 2025 Aug 6;27(5):1075–8. doi: 10.1002/epd2.70078 (PMC12574491; doi:10.1002/epd2.70078)
Supplement: Supplementary file 1 — Data S1. [file EPD2-27-1075-s001.zip › IIC MRP Supplemental Revision 2.docx]

Supplementary Data 1:


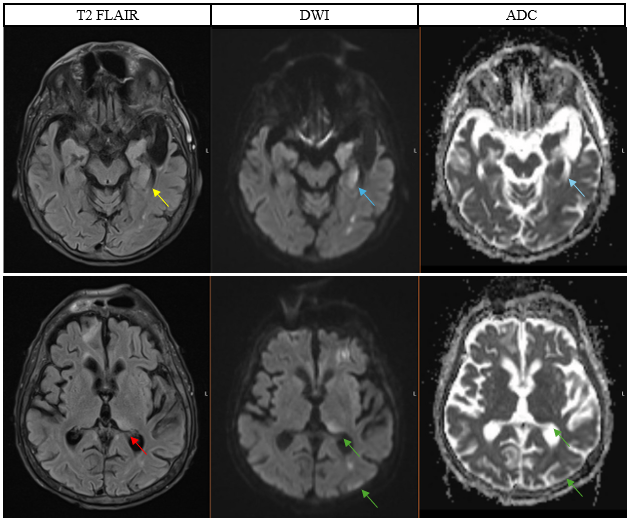


Figure 3: MRI brain (left to right, T2 Fluid-Attenuated Inversion Recovery (FLAIR), Diffusion-weighted Imaging (DWI), Apparent Diffusion Coefficient (ADC)) with post-ictal changes as seen by left temporal hyperintensity (yellow arrow) and diffusion restriction (blue arrow) and left thalamic/temporo-occipital hyperintensity (red arrow) and diffusion restriction (green arrows). Chronic left temporal encephalomalacia is present.

Supplemental 2:

LIMITATION

The patient had a seizure the evening after the MRI Perfusion scan, and ketamine was subsequently started. Despite escalation of treatment, the patient’s mental status did not improve, and he passed away after withdrawal of care. The presence of the patient's seizure later does not definitively rule that the IIC pattern was ictal but also does not definitively disqualify it. The pattern in itself increases seizure risk, which is known from multiple studies.

Supplemental 3:

The patient’s hospital course was complicated by distributive shock requiring three vasoactive medications that were not maintaining adequate blood pressure. The patient’s family opted to not escalate medical care, and he had a cardiac arrest and subsequently passed away.
