# Supplementary material for: Typical ictal pattern on MR perfusion scan for patients on the ictal–interictal continuum
Source: Epileptic Disord. 2025 Aug 6;27(5):1075–8. doi: 10.1002/epd2.70078 (PMC12574491; doi:10.1002/epd2.70078)
Supplement: Supplementary file 1 — Data S1. [file EPD2-27-1075-s001.zip › Epileptic Disorders IIC slides.pdf]

Typical ictal pattern on MR perfusion scan for patients on the ictal-interictal continuum

Melissa Huynh Mabry, M.D.[1], Hrishikesh Dadhich, M.D. [1], Zerrin Yetkin, M.D. [1, 2], Irfan Sheikh, M.D. [1, 3]

[1] Epilepsy Section, Department of Neurology, University of Texas Southwestern Medical Center, Dallas, Texas, USA
[2] Division of Neuroradiology, Department of Radiology, University of Texas Southwestern Medical Center, Dallas, Texas, USA
[3] Peter O'Donnell Jr. Brain Institute, University of Texas Southwestern Medical Center, Dallas, Texas, USA

Epileptic Disorders

Introduction

- Ictal-interictal continuum (IIC) consists of electrographic patterns that are concerning for seizures but do not meet criteria [1].
- IIC can be potentially ictal in certain cases and can warrant a therapeutic trial.

Case Report

- 59-year-old man with a past medical history of intraparenchymal hemorrhage presented for seizures and altered mental status concerning for status epilepticus.
- Initial EEG showed left temporal electrographic seizures, and the patient was started on levetiracetam, lacosamide, clobazam, and a midazolam infusion.
- After midazolam, EEG showed continuous 1 to 2 hz lateralized periodic discharges (LPDs) maximal at P7/O1, consistent with IIC (Figure 1).
- After 8 days in this pattern, MR perfusion was obtained and showed increased perfusion in the L temporo-occipital region (Figure 2) which was concerning for ongoing ictal activity.
- Despite escalation in treatment, his condition did not improve, and he passed away after withdrawal of care.

Figure 1:

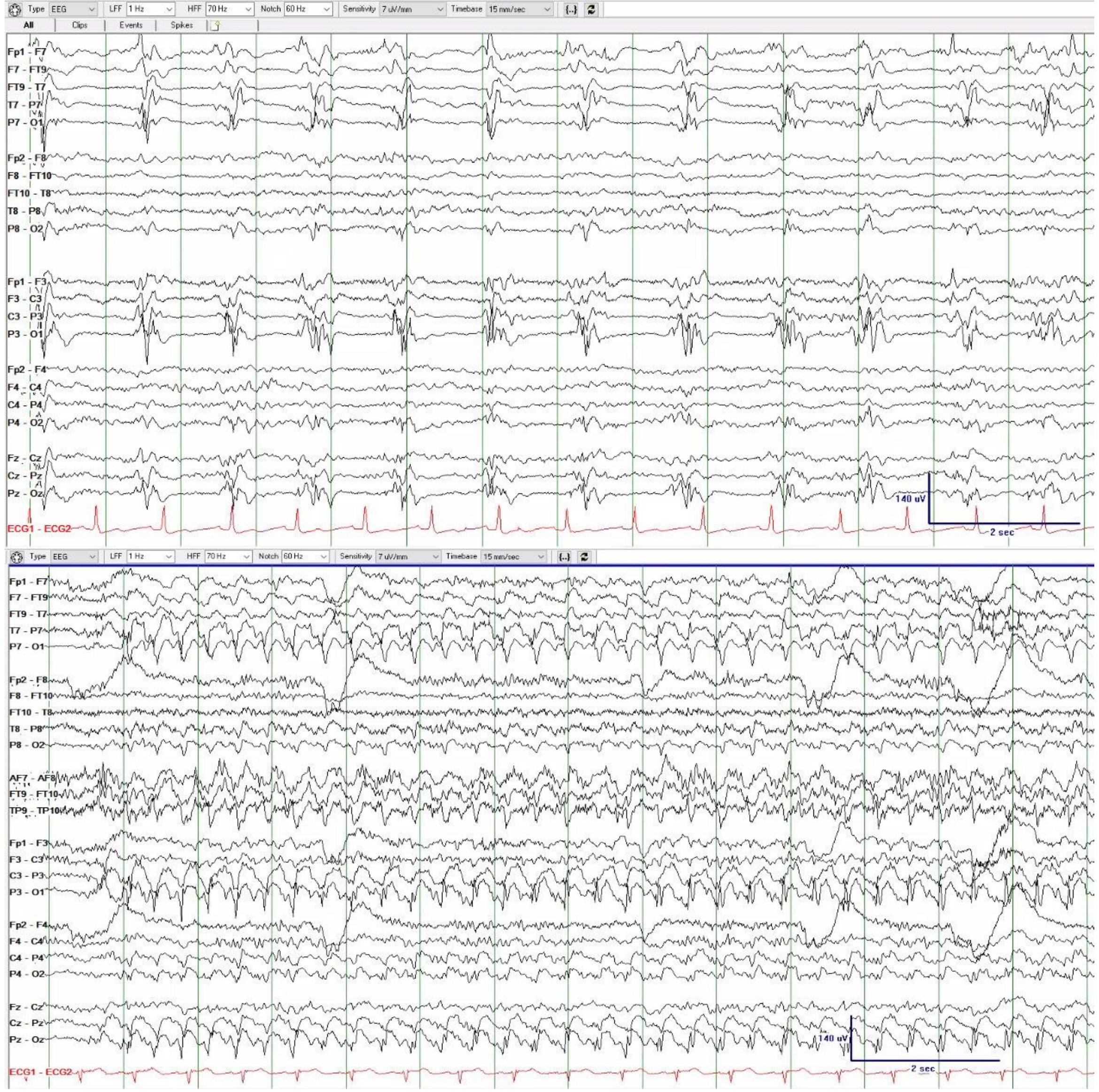

Figure 1. *EEG demonstrating left temporo-occipital LPDs (top panel) and electrographic seizure (bottom panel) thus highlighting the patient's background when in IIC versus when ictal. The electrographic seizure shown was recorded after the MR perfusion scan was performed.*

Figure 2:

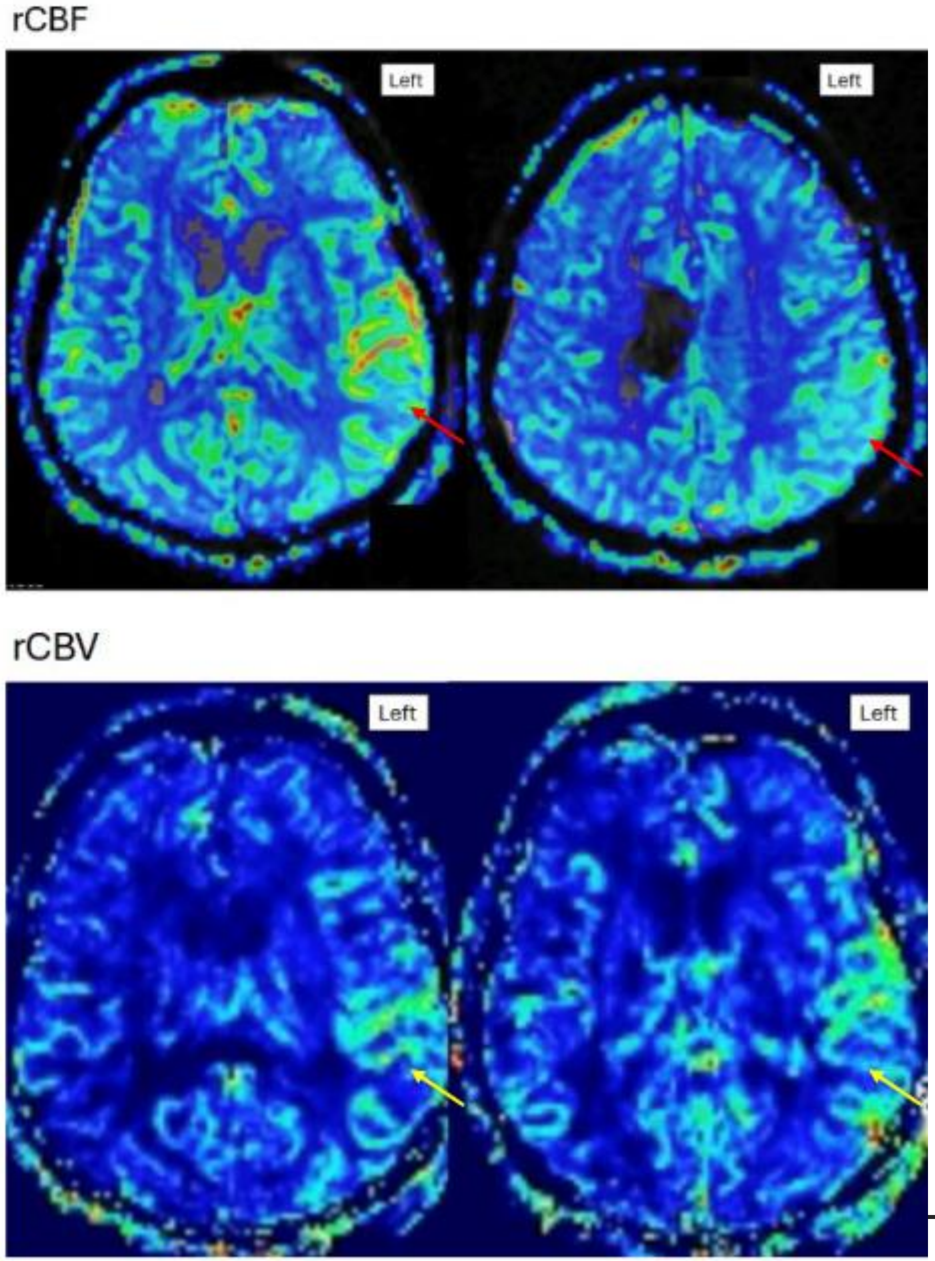

Figure 2: Dynamic susceptibility contrast-enhanced MR perfusion images demonstrate increased relative cerebral blood flow (rCBF; red arrows) and relative cerebral blood volume (rCBV; yellow arrows) of the left hemisphere. The increased perfusion, particularly in the left temporal lobe, indicated ongoing ictal activity despite the last electrographic seizure occurring eight days earlier.

Discussion

- The lateralized periodic discharges seen were between 1 and 2.5 hz with a duration much greater than 10 seconds and is considered part of IIC.
- The LPDS were present for 8 days before the MR perfusion was obtained.
- An MR perfusion will show a focal area of increased perfusion when there is ictal activity [2,3,4]
- 50% of patients with lateralized periodic discharges will not have restricted diffusion suggesting that they do not carry the same degree of neuronal injury as seizures [3,5].
  - Thus, it is hypothesized an IIC pattern with lateralized periodic discharges that are not ictal will not have increased perfusion on MR.
- A case report demonstrated that treatment of IIC patterns that correlated to increased perfusion on MR perfusion helped improve clinical outcomes [5].

Conclusion

- MR perfusion can help determine if an IIC pattern is more ictal than interictal and may alter clinical management.

References:

1 - Hirsch LJ, Fong MWK, Leitinger M, et al. N. American Clinical Neurophysiology Society's Standardized Critical Care EEG Terminology: 2021 Version. J Clin Neurophysiol. 2021 Jan 01;38(1):1-29.

2 - Kim TJ, Choi JW, Han M, et al. Usefulness of arterial spin labeling perfusion as an initial evaluation of status epilepticus. Sci Rep 2021;11(1):24218. doi: 10.1038/s41598-021-03698-7.

3 - Shimogawa T, Morioka T, Sayama T, et al. The initial use of arterial spin labeling perfusion and diffusion-weighted magnetic resonance images in the diagnosis of nonconvulsive partial status epileptics. Epilepsy Res 2017;129:162-173. doi: 10.1016/j.eplepsyres.2016.12.008.

4- Kim SE, Lee BI, Shin KJ, Ha SY, Park J, Park KM, Kim HC, Lee J, Bae SY, Lee D, Kim SE. Characteristics of seizure-induced signal changes on MRI in patients with first seizures. Seizure. 2017May;48:62-68.

5- Venkatraman A, Khawaja A, Bag AK, Mirza M, Szaflarski JP, Pati SBB. Perfusion MRI Can Impact Treatment Decision in Ictal-Interictal Continuum. J Clin Neurophysiol. 2017 Jul;34(4):e15-e18.
