# Supplementary material for: Typical ictal pattern on MR perfusion scan for patients on the ictal–interictal continuum
Source: Epileptic Disord. 2025 Aug 6;27(5):1075–8. doi: 10.1002/epd2.70078 (PMC12574491; doi:10.1002/epd2.70078)
Supplement: Supplementary file 2 — Data S2. [file EPD2-27-1075-s002.docx]

**TEST YOURSELF**

**Answers:**

**1. Answer: C.**

Per the American Clinical Neurophysiology Society's Standardized Critical Care EEG Terminology: 2021 version, IIC can be any periodic discharge that averages >1.0 hz and ≤2.5 hz over 10 s (>10 and ≤25 discharges in 10 s)

**2. Answer: B**

MR perfusion will show increased cerebral blood flow and volume in the seizure focus during the ictal phase due to increased metabolic demand

**3. Answer: A**
